# CDX2 Stimulates the Proliferation of Porcine Intestinal Epithelial Cells by Activating the mTORC1 and Wnt/β-Catenin Signaling Pathways

**DOI:** 10.3390/ijms18112447

**Published:** 2017-11-18

**Authors:** Hong-Bo Fan, Zhen-Ya Zhai, Xiang-Guang Li, Chun-Qi Gao, Hui-Chao Yan, Zhe-Sheng Chen, Xiu-Qi Wang

**Affiliations:** 1 College of Animal Science/Guangdong Provincial Key Laboratory of Animal Nutrition Regulation/National Engineering Research Center for Breeding Swine Industry, South China Agricultural University, Guangzhou 510642, China; fanhb@stu.scau.edu.cn (H.-B.F.); zhai@stu.scau.edu.cn (Z.-Y.Z.); li.7924@osu.edu (X.-G.L.); cqgao@scau.edu.cn (C.-Q.G.); yanhc@scau.edu.cn (H.-C.Y.); 2 Department of Pharmaceutical Science, College of Pharmacy and Health Science, St. John’s University, Queens, NY 11439, USA; chenz@stjohns.edu

**Keywords:** caudal type homeobox, signaling pathway, cooperation, proliferation

## Abstract

Caudal type homeobox 2 (CDX2) is expressed in intestinal epithelial cells and plays a role in gut development and homeostasis by regulating cell proliferation. However, whether CDX2 cooperates with the mammalian target of rapamycin complex 1 (mTORC1) and Wnt/β-catenin signaling pathways to stimulate cell proliferation remains unknown. The objective of this study was to investigate the effect of CDX2 on the proliferation of porcine jejunum epithelial cells (IPEC-J2) and the correlation between CDX2, the mTORC1 and Wnt/β-catenin signaling pathways. CDX2 overexpression and knockdown cell culture models were established to explore the regulation of CDX2 on both pathways. Pathway-specific antagonists were used to verify the effects. The results showed that CDX2 overexpression increased IPEC-J2 cell proliferation and activated both the mTORC1 and Wnt/β-catenin pathways, and that CDX2 knockdown decreased cell proliferation and inhibited both pathways. Furthermore, the mTORC1 and Wnt/β-catenin pathway-specific antagonist rapamycin and XAV939 (3,5,7,8-tetrahydro-2-[4-(trifluoromethyl)]-4H –thiopyrano[4,3-d]pyrimidin-4-one) both suppressed the proliferation of IPEC-J2 cells overexpressing CDX2, and that the combination of rapamycin and XAV939 had an additive effect. Regardless of whether the cells were treated with rapamycin or XAV939 alone or in combination, both mTORC1 and Wnt/β-catenin pathways were down-regulated, accompanied by a decrease in CDX2 expression. Taken together, our data indicate that CDX2 stimulates porcine intestinal epithelial cell proliferation by activating the mTORC1 and Wnt/β-catenin signaling pathways.

## 1. Introduction

CDX2, a caudal-related homeobox gene coding for a homeodomain transcription factor, is an essential regulator of gut development and homeostasis. CDX2 conditional knockout mice failed to form a mature endoderm in their intestinal epithelia [1] and inactivation of CDX2 in intestinal stem cells (ISCs) re-specified their identity and fated towards gastric stem cells [2]. By transcriptionally modulating the expression of relevant target genes, CDX2 plays a crucial role in cell processes including proliferation, differentiation, migration and apoptosis [3,4].

Differences exist with regard to the effect of CDX2 on cell proliferation in different observations. Studies on cancer cells, including human colorectal carcinoma cells (HT-29) [5], human colon adenocarcinoma cells (Caco-2) [4] and gastric cancer cells (BGC-823) [6], demonstrated that CDX2 inhibits cell proliferation. However, studies on rat intestinal epithelial cells (IEC-6) [7], porcine intestinal epithelial cells (IPEC-1) [8], blastocysts [9], and mice ISCs [10] produced opposite results, accompanied by high expression of Heparin-binding epidermal growth factor (EGF) like growth factor and proliferating cell nuclear antigen (PCNA) [7]. Therefore, it is still unknown how CDX2 plays dual roles in different types of cells. To address this issue, new models and different angles are needed to investigate and account for the discrepancy.

The mammalian target of rapamycin complex 1 (mTORC1) signaling pathway is responsible for translation and in the control of cell growth and metabolism. The Wnt/β-catenin signaling pathway is indispensable to cell polarity, proliferation, differentiation and migration. Both the expression level and the level of phosphorylation of the relevant functional proteins in both pathways are important parameters for measuring the state of cell proliferation [11,12]. Yet, whether the mTORC1 and Wnt/β-catenin pathways play an antagonistic or stacking role in CDX2-mediated proliferation is unknown. The comprehensive study of the correlation between CDX2, the mTORC1 and Wnt/β-catenin is deficient [13,14,15]. Considering the dual roles of CDX2 in cell proliferation, it is necessary to probe into the function of the mTORC1 and Wnt/β-catenin pathways in CDX2-mediated normal intestinal epithelial cell proliferation.

To further elucidate the effect of CDX2 in normal intestinal epithelial cell proliferation and the relationship between CDX2, and the mTORC1 and Wnt/β-catenin pathways, we constructed two models, an overexpression model and a knockdown model of CDX2 in the porcine jejunum epithelial cell line, IPEC-J2. Antagonists specific to the mTORC1 or Wnt/β-catenin pathways were used to validate the results.

## 2. Results

### 2.1. Overexpression of CDX2 in IPEC-J2 Cells Increased Cell Proliferation

**Figure 1:**
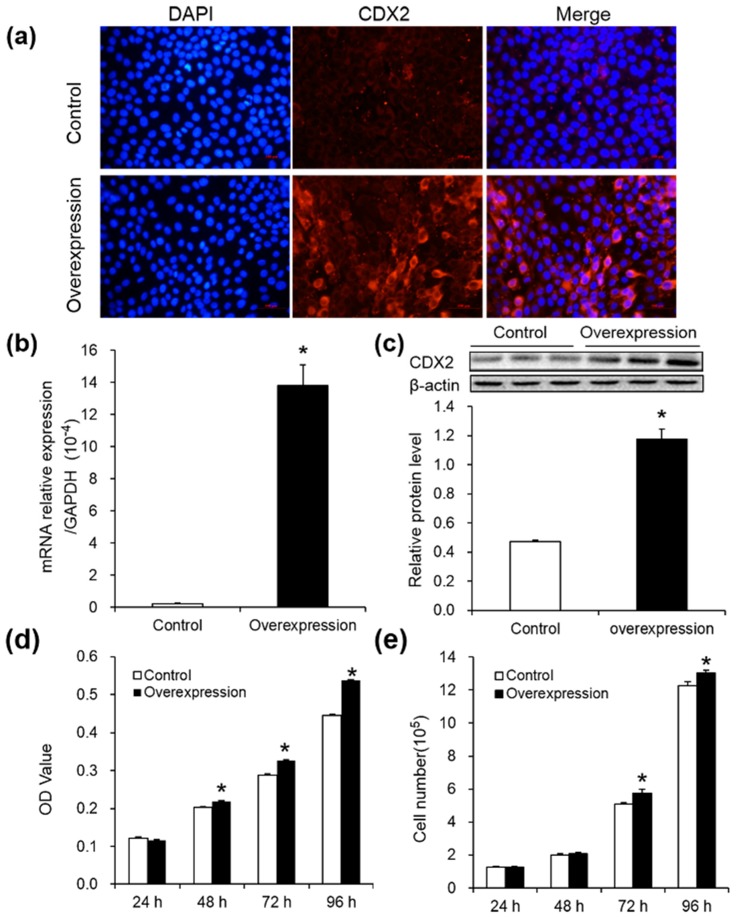
Caudal type homeobox 2 (CDX2) overexpressed in IPEC-J2 cells and increased cell proliferation. (**a**) Representative immunofluorescence images of control and overexpression groups 48 h after seeding, labeled with DAPI (blue) and CDX2 antibody (red) (200×). Scale bar: 100 μm. (**b,c**) *CDX2* mRNA abundance (*n* = 6) and protein expression (*n* = 3) in the control and overexpression group. AU: arbitrary unit; (**d,e**) the OD value and cell number were assessed by MTT assay (*n* = 20) and cell counting (*n* = 6), respectively. Control: control group; Overexpression: CDX2 overexpression group. Representative results of three independent experiments are shown as the mean ± SEM; * *p* < 0.05.

Immunofluorescence microscopy, real-time PCR and Western blot were performed to assess CDX2 overexpression in IPEC-J2 cells. Compared with the control group, CDX2 fluorescence signaling (Figure 1a), mRNA abundance (Figure 1b) and protein levels (Figure 1c) increased in the overexpression group, showing that the overexpressed cells were stably transfected with CDX2-pcDNA3.1+.

The MTT (3-(4,5-dimethylthiazol-2-yl)-2,5-diphenyltetrazolium bromide) assay and cell counting were used to evaluate the effect of CDX2 overexpression on IPEC-J2 cell proliferation. OD values (Figure 1d) and cell numbers (Figure 1e) were increased in the overexpression group.

### 2.2. CDX2 Overexpression Activated Both the mTORC1 and Wnt/β-Catenin Pathways in IPEC-J2 Cells

**Figure 2:**
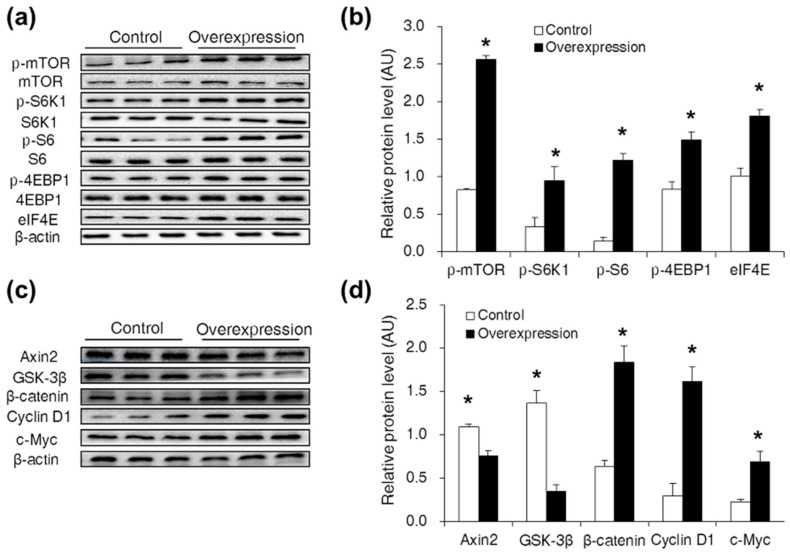
CDX2 overexpression activated both the mTORC1 and Wnt/β-catenin pathways. (**a,b**) Western blot analysis of the mTORC1 pathway activity after CDX2 overexpressed in IPEC-J2 cells with quantification (*n* = 3); (**c,d**) western blot of Wnt/β-catenin pathway related proteins after CDX2 overexpressed with quantification (*n* = 3). AU: arbitrary unit. Control: control group; Overexpression: CDX2 overexpression group. Representative results of three independent experiments are shown. Data are expressed as the mean ± SEM; * *p* < 0.05.

To measure the effect of CDX2 overexpression on the mTORC1 and Wnt/β-catenin pathways, Western blot analysis was used. Compared with the control group, levels of p-mTOR (Ser^2448^), p-S6K1 (Thr^389^), p-S6 (Ser^235^), p-4EBP1 (Thr^70^) and eIF4E were increased in the overexpression group (Figure 2a,b). Levels of Axin2 and GSK-3β were decreased, and levels of β-catenin, Cyclin D1 and c-Myc were increased in the overexpression group (Figure 2c,d).

### 2.3. CDX2 Knockdown in IPEC-J2 Cells Decreased Cell Proliferation

**Figure 3:**
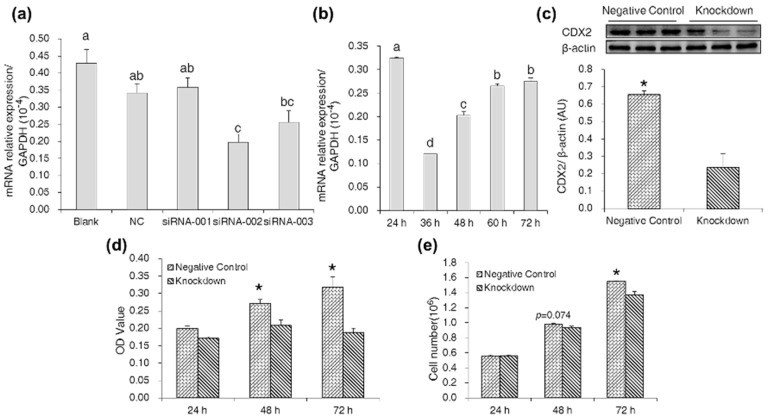
CDX2 knockdown in IPEC-J2 cells reduced cell proliferation. (**a**) The effect of three siRNAs on *CDX2* mRNA abundance was measured by real-time PCR 48 h post-transfection. Blank: control group; NC: negative control group; siRNA-001: CDX2-siRNA-001 group; siRNA-002: CDX2-siRNA-002 group; siRNA-003: CDX2-siRNA-003 group; (**b**) the effect of siRNA-002 transfection time on *CDX2* mRNA abundance was measured by real-time PCR. Data are expressed as the mean ± SEM (*n* = 6). The bars without same letters indicate a significant difference (*p* < 0.05); (**c**) siRNA-002 treatment reduced CDX2 protein expression compared with the negative control group. AU: arbitrary unit; (**d,e**) OD values and cell numbers were assessed by MTT assay (*n* = 20) and cell counting (*n* = 6), respectively. Negative Control: negative control group; Knockdown: CDX2-siRNA-002 group. Representative results of three independent experiments are shown. Data are expressed as the mean ± SEM; * *p* < 0.05.

By measuring CDX2 mRNA abundance at 48 h post-transfection with the three CDX2-siRNA, we found that siRNA-002 produced an optimal interference effect (Figure 3a). We also found CDX2 mRNA abundance in IPEC-J2 cells to be the lowest at 36 h post-transfection with siRNA-002 (Figure 3b). Compared with the negative control, Western blot analysis also showed a reduction in CDX2 expression in the knockdown group (Figure 3c).

The results of the MTT assay and cell counting showed that CDX2 knockdown decreased OD values (Figure 3d) and cell numbers (Figure 3e), respectively.

### 2.4. CDX2 Knockdown Inhibited Both the mTORC1 and Wnt/β-Catenin Pathways in IPEC-J2 Cells

**Figure 4:**
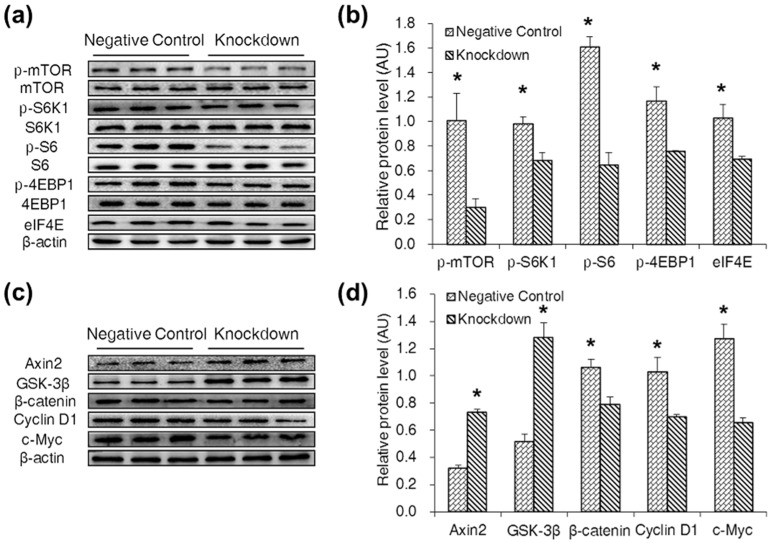
CDX2 knockdown inhibited both the mTORC1 and Wnt/β-catenin pathways. (**a,b**) Western blot analysis of the mTORC1 pathway activity after CDX2 knockdown in IPEC-J2 cells with quantification (*n* = 3); (**c,d**) western blot of Wnt/β-catenin pathway related proteins after CDX2 knockdown with quantification (*n* = 3). AU: arbitrary unit. Negative Control: negative control group; Knockdown: CDX2-siRNA-002 group. Representative results of three independent experiments are shown. Data are expressed as the mean ± SEM (*n* = 3); * *p* < 0.05.

Western blot was used to evaluate the effect of CDX2 knockdown on the mTORC1 and Wnt/β-catenin pathways. The result showed that levels of p-mTOR (Ser^2448^), p-S6K1 (Thr^389^), p-S6 (Ser^235^), p-4EBP1 (Thr^70^) and eIF4E were decreased in the knockdown group (Figure 4a,b). Levels of Axin2 and GSK-3β were increased, and levels of β-catenin, Cyclin D1 and c-Myc were decreased in the knockdown group (Figure 4c,d).

### 2.5. Specific Antagonists Decreased Cell Proliferation and the Protein Level of CDX2 in CDX2 Overexpressing IPEC-J2 Cells

**Figure 5:**
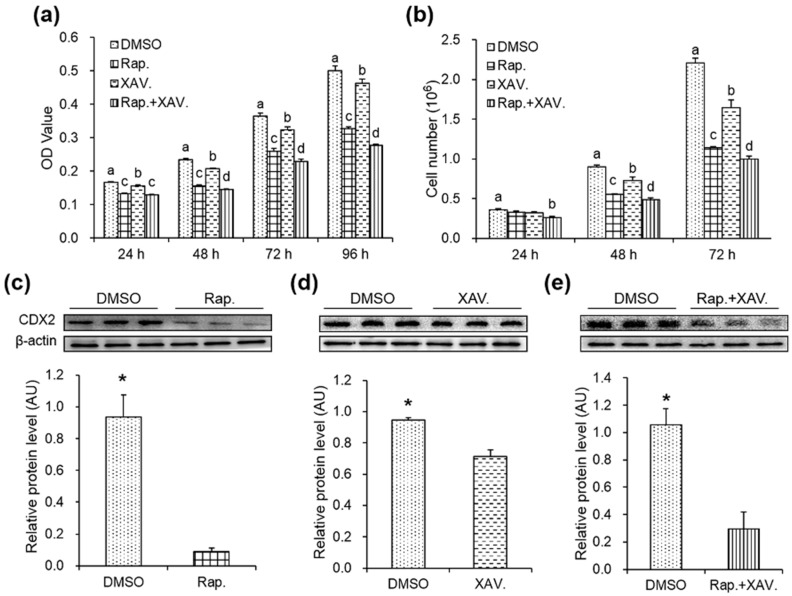
Specific antagonists decreased cell proliferation and the protein level of CDX2 in CDX2 overexpressed IPEC-J2 cells. (**a,b**) OD values and cell numbers were assessed by MTT assay (*n* = 20) and cell counting (*n* = 6), respectively; (**c**–**e**) protein levels of CDX2 were measured by Western blot after treated with rapamycin and XAV939 alone or in combination with quantification (*n* = 3). AU: arbitrary unit. DMSO: DMSO treatment group; Rap.: rapamycin treatment group; XAV.: XAV939 treatment group; Rap. + XAV.: combination of rapamycin and XAV939 treatment group. Representative results of three independent experiments are shown. Data are expressed as the mean ± SEM; bars without the same letter indicate a significant difference; * *p* < 0.05.

To validate the stimulatory effect of CDX2 on cell proliferation, the CDX2 overexpressing IPEC-J2 cells were treated with specific antagonists. The OD values from the MTT assay (Figure 5a) and cell numbers from the cell counting (Figure 5b) were used to evaluate the effect of the mTORC1 and Wnt/β-catenin pathway-specific antagonist rapamycin and XAV939 on cell proliferation of CDX2 overexpressing IPEC-J2 cells. The results showed that rapamycin and XAV939 decreased cell proliferation compared with the DMSO group, and the combination of rapamycin and XAV939 resulted in a synergistic decrease in cell proliferation. Moreover, rapamycin reduced cell proliferation more significantly than XAV939, suggesting a more essential role for the mTORC1 pathway in CDX2-mediated cell proliferation. These data indicated that both the mTORC1 and Wnt/β-catenin pathways participated in CDX2-mediated cell proliferation.

Rapamycin and XAV939, supplemented both individually and in combination, decreased CDX2 expression (Figure 5c–e). However, no synergistic effect was observed in the combination group.

### 2.6. Specific Antagonists Inhibited Both the mTORC1 and Wnt/β-Catenin Pathways in CDX2 Overexpressing IPEC-J2 Cells

**Figure 6:**
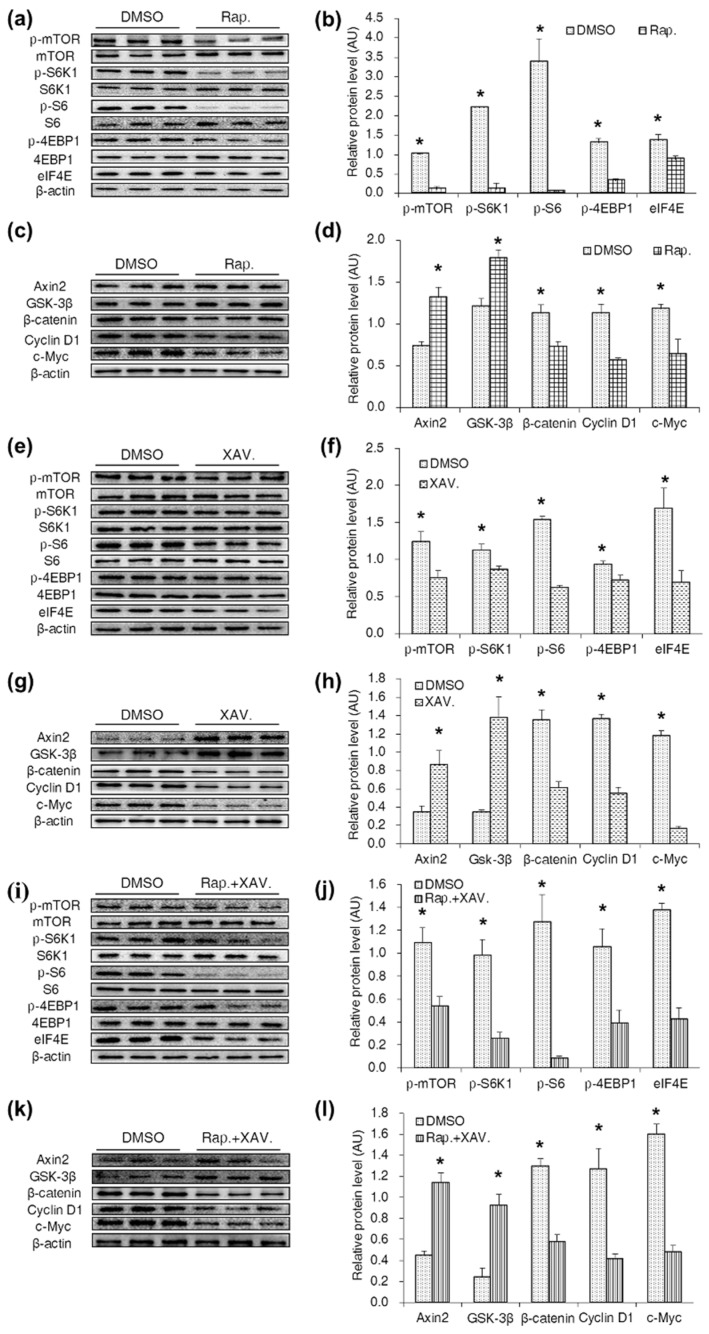
Specific antagonists inhibited both the mTORC1 and Wnt/β-catenin pathways in CDX2 overexpressed IPEC-J2 cells. (**a**–**d**) Western blot analysis of the mTORC1 and Wnt/β-catenin pathways activity after treated with rapamycin in CDX2 overexpressed IPEC-J2 cells with quantification (*n* = 3); (**e**–**h**) western blot of the mTORC1 and Wnt/β-catenin pathways related proteins after treated with XAV939 in CDX2 overexpressed IPEC-J2 cells with quantification (*n* = 3); (**i**–**l**) protein levels of the mTORC1 and Wnt/β-catenin pathways were measured by Western blot after treated with rapamycin and XAV939 in combination with quantification (*n* = 3). AU: arbitrary unit. As rapamycin and XAV939 were dissolved in DMSO, the DMSO-treated cell strain was the control group. DMSO: DMSO treatment group; Rap.: rapamycin treatment group; XAV.: XAV939 treatment group; Rap. + XAV.: combination of rapamycin and XAV939 treatment group. Representative results of three independent experiments are shown. Data are expressed as the mean ± SEM (*n* = 3); * *p* < 0.05.

To validate the stimulating effect of CDX2 on mTORC1 and Wnt/β-catenin pathways, specific antagonists were supplemented. The mTORC1 and Wnt/β-catenin pathway-related proteins were identified and quantified by Western blot. The results showed that regardless of whether rapamycin or XAV939 was supplemented alone or in combination, the levels of p-mTOR (Ser^2448^), p-S6K1 (Thr^389^), p-S6 (Ser^235^), p-4EBP1 (Thr^70^) and eIF4E decreased. Similarly, the levels of Axin2 and GSK-3β increased, and the levels of β-catenin, Cyclin D1 and c-Myc decreased (Figure 6a–l) in the Wnt/β-catenin pathway after treatment with one or both of the drugs.

## 3. Discussion

CDX2 acts as a biomarker for gastrointestinal cancer and inhibits the proliferation of different types of cancer cells [16,17,18]. However, the functional effects of CDX2 on the proliferation of normal intestinal epithelial cells are diverse. Studies on normal intestinal epithelial cells such as IEC-6 [7], IPEC-1 [8], blastocysts [9], and ISCs [10] revealed that CDX2 stimulates cell proliferation. In addition, porcine milk-derived exosomes increase CDX2 protein expression and IPEC-J2 cell proliferation simultaneously, suggesting that cell proliferation and CDX2 expression do not conflict [19]. In this study on IPEC-J2 cells, CDX2 overexpression increased cell proliferation and CDX2 knockdown decreased cell proliferation. These results collectively demonstrate that CDX2 stimulated proliferation in IPEC-J2 cells.

How does the conflicting function of CDX2, either stimulating or inhibiting cell proliferation, exist in different cells? The varied target genes of CDX2 may account for this dual function. The study on ISCs and villus cells (VCs) demonstrated that CDX2 occupies hundreds of genomic sites in ISCs and thousands of additional sites in VCs [10]. Moreover, in human intestinal epithelial crypt (HIEC) cells, DNA microarray analysis revealed 237 genes were modulated by CDX2 [20], and 604 peaks identified as potential CDX2 binding sites in Caco-2 cells [21]. Additionally, CDX2 behaves as a transactivator in certain kinds of cells, it may directly activate or repress genes in other cells [10]. Hence, in view of its roles in ISCs and VCs, and the different target genes in HIEC and Caco-2 cells, the stimulating or inhibiting effect of CDX2 on cell proliferation is explicable. Further study exploring the regulators of CDX2 is necessary to account for the different CDX2 occupancy and diverse effects on cell proliferation.

In consideration of the conflicting role of CDX2 on cell proliferation and to obtain further insight into its stimulating effect, we measured the expression of proteins in the mTORC1 and Wnt/β-catenin pathways. Firstly, the present study found that phosphorylated proteins in the mTORC1 pathway were up-regulated in the overexpression group and were down-regulated in the knockdown group, which suggested that CDX2 might activate the mTORC1 pathway in IPCE-J2 cells. However, few studies have shown that mTORC1 activity may be decreased by CDX2. A study on CDX2 heterozygous knockout mice demonstrated CDX2 haploinsufficiency led to an increase in mTOR kinase activity [22] and CDX2 overexpression in gastric cancer cells induced an increase of *mTOR* mRNA and protein expression [15]. The mTORC1 pathway is a central regulator of cell proliferation [12]. Given the dual role of CDX2 in cell proliferation, the activating effect on the mTORC1 pathway in IPEC-J2 cells might be reasonable.

Similarly, no conclusions about the activating or inhibiting effect of CDX2 on the Wnt/β-catenin pathway can be drawn. In human colon cancer cells, CDX2 limited cell proliferation and the Wnt/β-catenin signaling pathway by binding β-catenin and disrupting the β-catenin-TCF complex [13]. In contrast, chromatin immunoprecipitation (ChIP) performed on mice embryos demonstrated that CDX2 binds to the Wnt3a promoter, which acts as an agonist for the Wnt/β-catenin pathway, suggesting that CDX2 excites the Wnt/β-catenin pathway [14]. Likewise, the conventional Wnt/β-catenin pathway was activated in the overexpression group and repressed in the knockdown group in this study. Considering the essential role of the Wnt/β-catenin pathway in cell proliferation, these results demonstrate that CDX2 might be an activator for the Wnt/β-catenin pathway in IPEC-J2 cells.

Our results suggest that both the mTORC1 and Wnt/β-catenin pathways play a part in CDX2-mediated cell proliferation. While it is necessary to investigate which pathway is more important and if there is a synergistic effect on cell proliferation, it is noteworthy that treatment with rapamycin alone, XAV939 alone, or with a combination of both caused a significant decrease in the proliferation of CDX2 overexpressing IPEC-J2 cells, with a synergistic effect in the cells treated with both drugs. Furthermore, rapamycin treatment led to a more significant reduction in cell proliferation than the XAV939 treatment. Moreover, CDX2 expression was reduced in each treatment, which is in agreement with previously published data that identifies CDX2 as a direct Wnt target during hindgut differentiation [23]. Taken together, these results show that under the regulation of CDX2, the mTORC1 and Wnt/β-catenin pathways cooperated in stimulating cell proliferation, with the mTORC1 pathway playing a more critical role. Further, CDX2 expression depended on signal transduction in the mTORC1 and Wnt/β-catenin pathways.

Studies have shown that the mTORC1 and Wnt/β-catenin pathways positively regulate each other. Inoki et al., (2006) reported that GSK3 (glycogen synthase kinase) participates in the activation of TSC2 (tuberous sclerosis s) to inhibit mTORC1 activity [24]. β-catenin knockdown in colon cancer cell lines reduces the mTORC1 level and thereby inhibits mTORC1 signaling [25]. Conversely, kinases such as active Akt and p70S6K can phosphorylate GSK-3β at Ser9, resulting in the activation of the Wnt/β-catenin signaling pathway [26]. The level of β-catenin was markedly decreased by the mTORC1 antagonist rapamycin in hepatoma G2 (HepG2) cells and Hep3B cells [27]. The present study shows that, supplementation with either rapamycin or XAV939 resulted in the simultaneous inhibition of the mTORC1 and Wnt/β-catenin pathways. Therefore, our result might suggest crosstalk between the mTORC1 and Wnt/β-catenin pathways in IPEC-J2 cells.

**Figure 7:**
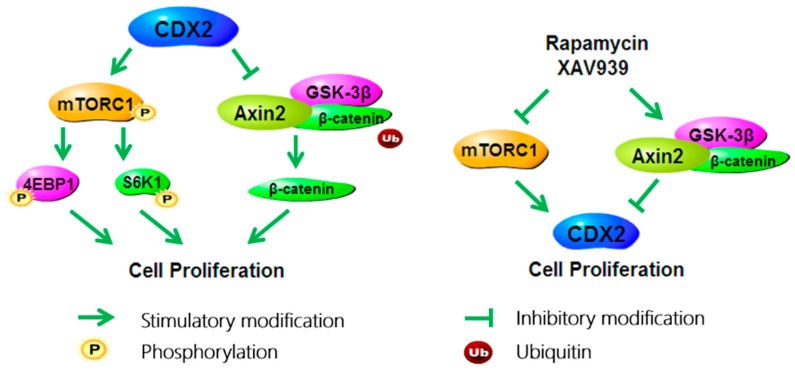
In IPEC-J2 cells, CDX2 promotes cell proliferation via activating the mTORC1 and Wnt/β-catenin pathways; specific antagonists of the mTORC1 and Wnt/β-catenin pathways, namely rapamycin or XAV939 respectively, decrease cell proliferation and inhibit both pathways simultaneously.

In summary, the present study illustrates the promoting effects of CDX2 on IPEC-J2 cell proliferation, activating the mTORC1 and Wnt/β-catenin pathways. Moreover, the study uncovers the mutual regulation among the mTORC1 and Wnt/β-catenin pathways and CDX2 (Figure 7).

## 4. Materials and Methods

### 4.1. Cell Culture

IPEC-J2 cells, kindly provided by Dr. Yu-long Yin (University of Chinese Academy of Sciences), were cultured in DMEM/F12 medium (Thermo, 12400-024, Waltham, MA, USA) supplemented with 5% fetal bovine serum (Gibco, 10099-141, Waltham, MA, USA), 1% penicillin/streptomycin, 5 μg/L Insulin-Transferrin-Selenium (ScienCell, 0803, Carlsbad, CA, USA) and 5 μg/L epidermal growth factor (ScienCell, 105-04) at 37 °C in 5% CO_2_. Medium was changed every two days [28]. Treatments including dimethyl sulfoxide (DMSO, 0.2%, Sigma-Aldrich, St. Louis, MI, USA), rapamycin (20 nm, 53123-88-9, Alexis, Farmingdale, NY, USA), XAV939 (3,5,7,8-tetrahydro-2-[4-(trifluoromethyl)]-4H –thiopyrano[4,3-d]pyrimidin-4-one, 10 μM, Selleck, Houston, TX, USA) and RNA interference were performed 24 h after seeding. All experiments were performed on cells at passages 10–20.

### 4.2. RNA Interference

Three siRNAs targeting *CDX2* and an unrelated negative control siRNA were designed and synthesized, and then purified via high performance liquid chromatography (RiboBio, Guangzhou, China) (Table S1). 24 h prior to transfection, cells were plated into a 6-well plate at 30–50% confluence. siRNAs (50 nM) were transfected into cells according to the manufacturer’s instructions. The unrelated control siRNA was comprised as the negative control.

### 4.3. Immunofluorescence Microscopy

pcDNA3.1+ and CDX2-pcDNA3.1+ were stably transfected into cells. Transfected cells were seeded at 1 × 10^4^ cells/mL into 96-well culture plates (Corning, #3599, New York, NY, USA) and fixed with 4% paraformaldehyde for 30 min. Cells were then permeabilized with 0.3% Triton X-100 for 15 min and blocked in a protein solution (Dako, Santa Clara, CA, USA) for 20 min. The primary antibody, anti-CDX2 (1:500 in antibody diluent; Cell Signaling Technology, Danvers, MA, USA), was incubated with the cells for 2 h at room temperature. Secondary staining was performed with Cy3-conjugated antibodies (1:200 in antibody diluent; Sangon Biotech, Shanghai, China) and incubated at room temperature for 1 h. Nuclei were stained with 4’6-diamidino-2-phenylindole (DAPI, 1:1000 in PBS; Sigma) for 5 min at room temperature. Fluorescence signals were observed with a fluorescence microscope (NIS-Elements, Nikon, Melville, NY, USA). Three independent experiments were performed.

### 4.4. Real-Time PCR

Using a Stratagene Mx3005P qPCR system (Agilent Technologies, Santa Clara, CA, USA) and the SYBR Green Real-Time PCR Master Mix (Toyobo, Osaka Prefecture, Japan), mRNA expression (*n* = 6) was determined. Specific primer pairs used are detailed in Table S2. A melting curve analysis was conducted to confirm the specificity of each product, and product sizes were verified using ethidium bromide-stained, 1.5% agarose gels in Tris acetate-EDTA buffer. Quantitative data were obtained using the 2^−ΔΔ*C*t^ method. The experiment was performed in triplicate.

### 4.5. MTT Assay

Cells were seeded in 96-well plates at 1 × 10^4^ cells/mL. After incubating the cells in 5 mg/mL 3-(4,5-dimethylthiazol-2-yl)-2,5-diphenyltetrazolium bromide (MTT, Sigma) for 4 h, crystals were then dissolved in DMSO. Optical density (OD) values were evaluated in an ELISA reader (iMark, Bio-Rad, Hercules, CA, USA) at a wavelength of 490 nm. Three independent experiments with 20 samples per treatment were conducted to confirm the result.

### 4.6. Cell Counting Assay

Cells were seeded in 6-well plates at 1 × 10^5^ cells/mL. After washing twice with PBS, cells were detached with 0.25% trypsin (Sigma) for 5–8 min at 37 °C, and then blocked with an equal volume of growth medium. The number of viable cells was determined using a automate cell counter (Countstar BioTec, Shanghai, China). Three independent experiments were conducted.

### 4.7. Western Blot Analysis

Cells were lysed in RIPA buffer, containing 1% Triton-X, 20 mmol/L Tris-HCl (pH = 7.4), 5 mmol/L EDTA and 1 mmol/L phenylmethylsulfonyl fluoride (PMSF), and then followed by 14,000× *g* centrifugation at 4 °C for 15 min. Protein concentrations were determined by a BCA Protein Assay Reagent Kit (Thermo Fisher Scientific). Proteins (10 μg) were separated on 10% SDS/PAGE gels, and transferred onto PVDF membranes (Millipore, Burlington, MA, USA). After blocking with bovine serum albumin (BSA), the membranes were incubated with a primary antibody. Anti-CDX2 (#12306), anti-mTOR (#2972), anti-phospho-mTOR (Ser^2448^, #5536), anti-S6K1 (#9202), anti-phospho-S6K1 (Thr^389^, #9205), anti-S6 (#2708), anti-phospho-S6 (Ser^235/236^, #9234), anti-4EBP1 (#9644), anti-phospho-4EBP1 (Thr^70^, #2855), and anti-c-Myc (#5605) antibodies were obtained from Cell Signaling Technology; anti-GSK-3β (sc-9166) and anti-Cyclin D1 (sc-753) were purchased from Santa Cruz Biotechnology; anti-β-catenin (ab6302), anti-Axin2 (ab109307) and anti-eIF4E antibodies were obtained from Abcam; and anti-β-actin (AP0060), anti-rabbit IgG (BS13278) and anti-goat IgG (BS30503) antibodies were obtained from Bioworld Technology. Proteins were visualized using the Beyo ECL Plus chemiluminescence detection kit (Beyotime Institute of Biotechnology, Shanghai, China). ECL signals were detected by a FluorChem M imaging system (Protein Simple, San Jose, CA, USA). Band density was calculated using Image Analysis Software (Tanon, Shanghai, China). Results were normalized to β-actin or the total level.

### 4.8. Statistics

Data are expressed as the mean ± SEM. Student’s *t*-test was conducted to determine the differences between 2 groups using SAS (version 9.2, SAS Institute Inc., Cary, NC, USA), and Duncan’s multiple-range test to determine differences among groups. Differences were considered statistically significant when *p* < 0.05.

## Supplementary Materials

Supplementary materials can be found at www.mdpi.com/1422-0067/18/11/2447/s1.

## Abbreviations

CDX2	caudal type homeobox 2
mTORC1	mammalian target of rapamycin complex 1
IPEC-J2	porcine jejunum epithelial cell line
ISCs	intestinal stem cells
EGF	epidermal growth factor
PCNA	proliferation cell nuclear antigen
siRNA	small interfering RNA
Axin2	axis inhibition protein 2
GSK-3β	glycogen synthase kinase 3β
eIF4E	eukaryotic initiation factor 4E
S6K1	ribosomal protein S6 Kinase-1
4EBP1	eukaryotic initiation factor 4E binding protein 1
VCs	villus cells
HIEC	human intestinal epithelial crypt cell
