# Supplementary material for: CDX2 Stimulates the Proliferation of Porcine Intestinal Epithelial Cells by Activating the mTORC1 and Wnt/β-Catenin Signaling Pathways
Source: Int J Mol Sci. 2017 Nov 18;18(11):2447. doi: 10.3390/ijms18112447 (PMC5713414; doi:10.3390/ijms18112447)
Supplement: Supplementary file 1 [file ijms-18-02447-s001.pdf]

Table S1. Sequences of chemically synthesized CDX2-siRNAs

Name	Type	Sequence
siRNA-001-sence	RNA	5′ CAUCACCAUUCGGAGAAAG dTdT 3′
siRNA-001-antisense	RNA	3′ dTdT GUAGUGGUAAGCCUCUUUC 5′
siRNA-002-sence	RNA	5′ GACAAGGACGUGAGCAUGU dTdT 3′
siRNA-002-antisense	RNA	3′ dTdT CUGUUCCUGCACUCGUACA 5′
siRNA-003-sence	RNA	5′ ACAGUCGCUACAUCACCAU dTdT 3′
siRNA-003-antisense	RNA	3′ dTdT UGUCAGCGAUGUAGUGGUA 5′

Table S2. Primers used for quantitative real-time PCR

Genes	Primers	Sequence (5′-3′)	Product Size (bp)
CDX2	Sense	GTCGCTACATCACCATTCGG	110 bp
	Antisense	GATTTTCCTCTCCTTCGCTCT	
GAPDH (NM_001206359.1)	Sense	AGGTCGGAGTGAACGGA	145 bp
	Antisense	TGGGTGGAATCATACTGG	
